# Influence of Perceived Parent and Peer Endorsement on Adolescent Smoking Intentions: Parents Have More Say, But Their Influence Wanes as Kids Get Older

**DOI:** 10.1371/journal.pone.0101275

**Published:** 2014-07-03

**Authors:** Francesca Scalici, Peter J. Schulz

**Affiliations:** Institute of Communication and Health, Università della Svizzera italiana, Lugano, Switzerland; The University of Queensland, Australia

## Abstract

**Purpose:**

The aim of the study is to investigate how adolescents' perception of parents' and peers' smoking approval influences adolescent smoking intention, and how age affects this influence in a Swiss sample of adolescents. To know the influence of age can help to develop specific prevention programs tailored to the age groups needs.

**Method:**

in a cross sectional survey, students aged between 11 and 14 from public and private middle schools in the Italian region of Switzerland (Ticino) answered questions on smoking habits, parents' and peers' approval and intention to smoke.

**Results:**

peers' and parents' approval significantly influence students' smoking intention, and students' age significantly moderates this relation: the effect of parents' approval decreases for older adolescents, while the effect of peers' approval increases with age. No difference is found between girls and boys, while non-Swiss are more likely to smoke than Swiss students.

**Conclusions:**

as literature suggests, results evidence the role parents play during early adolescence. Prevention programs targeting parent-child communication in early adolescence for preventing children's tobacco consumption are strongly supported.

## Introduction

The increase of young smokers in Switzerland has become a serious problem. The last international study promoted by «Health Behavior in School-aged Children» (HBSC) shows that after a decrease in 2006, the number of Swiss students who smoke at least once a week has started to increase again in 2010. 10.5% of girls and 13.2% of boys, aged 15, smoke every day, a difference not found significant in the study that unearthed it [1]. 23.7% of middle schools students of the Italian region of Switzerland (Ticino) aged between 11 and 14 years have already smoked a cigarette. The survey “Tabacco e giovani 2012”, conducted by the University of Lugano (Università della Svizzera italiana) in collaboration with the Swiss Non-Smokers' Association and the Cantonal School Department, shows that 26.2% of boys and 20.7% of girls, 11–14 years old, have already smoked once. Among those who had, 20.6% were, at the time of survey, heavy regular smokers, 15.4% light smokers and 57.9% non-smokers with smoking experience (students who have tried smoking at least once but are not currently smokers). The percentage of heavy regular smokers and light smokers among girls is higher than boys in this survey.

Different factors like cultural, social and economic conditions influence adolescents' substance consumption and initiation. The social context the adolescent lives in is a strong determinant of substance use [2]. For that reason, social norms are often associated with adolescents' substance use [3], [4]. In the context of our study, we consider norms as defined by the psychologists: as features of small groups [5]. Research on social norms and adolescents' substance consumption distinguishes descriptive and subjective norms (i.e. injunctive norms) and evaluates their impact on adolescents' substance use [3], [4], [6], [7]. While descriptive norms give information about the perception of other people's behaviors, injunctive norms determine the perceived acceptance of a social behavior by other people [6]. In the area of adolescent substance use, both parents' and peers' injunctive norms (i.e. perceived parents' and peers' approval/disapproval) and parents' and peers' descriptive norms (i.e. perceived parents' and peers' substance use) are associated with adolescents' substance use [3], [4].

In adolescence, peer and parental social norms, descriptive and injunctive, play a key role in decision making on engaging in a behavior, and both norms and both referents have effects on adolescents' smoking behaviors [7], [8], [9], [10], [11], [12], [13]. There is no complete agreement about the impact of the two kinds of norms on adolescents' substance use. For a strand of literature, parents' and peers' injunctive norms clearly predict adolescents' alcohol and marihuana use and smoking behavior and intention [8], [9], [13], [14], [15], [16], [17], [18], [19], [20], and they are more influential than descriptive norms [8], [9], [21]. Adolescents are less likely to smoke if they perceive their parents and peers disapprove of smoking or react negatively to it [8], [9], [16], [17], [18]. Otherwise, studies evidence stronger associations of peers' and parents' descriptive norms (as compared to injunctive) with adolescents' alcohol [4], [22], [23], marijuana [24] and tobacco consumption [25], [26].

There is no agreement concerning which of the referents, parents or peers, exert the stronger influence on adolescents' smoking behaviors. Some comparisons between parents' and peers' social norms evidence no difference in impact [10], [11] while another strand of the literature shows a stronger influence of peers [12]. To conclude, the investigated literature suggests both referents (parents and peers) and both norms (subjective and descriptive) have effects on smoking behavior, with parents' and peers' descriptive norms predicting actual substance use [9] while parents' and peers' subjective norms (i.e. injunctive norms) more strongly affect the intention [7], [9].

Based on these studies, and considering that intention is a good predictor of smoking behaviors [11], [21], [27], we aim to investigate how parents' and peers' injunctive norms influence adolescents' smoking intention. Although many studies approached these topics, few of them have focused on the perception of injunctive norms across different age groups in a Central European sample of adolescents. We replay Sawyer and Stevenson's [7] model of the influence of parents' and peers' approval on the intention to use drugs across different age groups for a particular population, Ticino middle school students, and for a specific substance, tobacco. The large sample size, the inclusion of both peers' and parents' norms, and the examination of injunctive norms in a Central European sample of adolescents, represent the major strengths of this paper. The findings suggest tobacco prevention programs and policies targeting parent-child communication that is: aiming at teaching parents to better communicate with their children about tobacco use.

### Hypotheses and Research Question

Literature suggests the following hypotheses:

**H1.** Students who think their parents approve of adolescent smoking will show a higher intention to smoke than students who think their parents disapprove.

**H2.** Students who think their peers approve of adolescent smoking will show a higher intention to smoke than students who think their peers disapprove.

**RQ1.** Which will affect intention to smoke more, parents' approval or peers' approval?

This research question hides our expectation that the effect of parents' and peers' approval will be moderated, in opposing direction, by student age, as specified in:

**H3.** Older students will show a weaker effect of perceived parents' approval on intention to smoke than younger students.

**H4.** Older students will show a stronger effect of perceived peers' approval on intention to smoke than younger students.

## Method

At the time of data collection, in 2011, the University of Lugano had not yet established an IRB (this was done in Summer 2013). Before the schools became directly involved, the cantonal Department of Education reviewed the study protocol and the questionnaire and provided to the University a formal declaration of agreement, which allowed us to collect data in schools. No parental consent was required for this study due to the cantonal approval.

### Data collection and sample description

Data was collected via a cross sectional survey conducted from October 2011 to January 2012 by the Institute of Communication and Health (Università della Svizzera italiana) in collaboration with the Swiss Non-smokers' Association in Ticino and the Cantonal School Department (Survey S1). Pre-tests were carried out in May and June 2011, when different versions of the questionnaire were administered four times to 10 students from the first and second class, and to 8 students attending the third and fourth one. After every test run, the questionnaire was adapted and tested again. The final questionnaire considered all the findings from the test runs.

The sample includes students from all 42 public and private middle schools of Ticino. Out of a total of 598 classes, 285 were randomly selected to participate in the survey, namely 69 for the first grade, 69 for the second grade, 73 for the third grade and 74 for the fourth grade. The 285 classes had 5890 students (Table 1). The school director personally informed students about the survey. On the day of the survey, teachers described the study protocol to the students and asked for their written consent. Students who agreed to participate received the paper and pencil questionnaire and proceeded to completing it.

**Table 1:** Sample description.

	N (5657)	%
**Gender**		
Male	2824	49.9
Female	2772	49.0
**Nationality**		
Swiss	4182	73.9
Italian	506	8.9
Others	710	12.6
**Grade**		
First (11years)	1367	24.2
Second (12 years)	1361	24.1
Third (13 years)	1384	24.5
Fourth (14 years)	1486	26.3

We received 5657 questionnaires correctly completed, that represents 96.0% of the sample (response rate) and 46.3% of the total population of Ticino middle school students (*Total N*: 12210). Anonymity was guaranteed insofar as the children, once they had completed the questionnaire, put the questionnaire in a covered box, which was afterwards handed over to the researchers. Filling out the questionnaire lasted around 25 minutes.

### Measures

The administrated questionnaire includes several measures concerning tobacco consumption and related behaviors, not all of which are used in the present analysis (Survey S1). The following measures are employed:

perceived parents' approval was measured with one item, “What do your parents think about adolescents smoking?” Answers were recorded as 1 “approve,” 2 “neutral” and 3 “do not approve”.

Perceived peers' approval was measured with one item, “What do your friends think about their peers smoking?” Answers were recorded as 1 “approve,” 2 “neutral” and 3 “do not approve”.

Intention to smoke was measured with one item, “Now, think about yourself, would you like to smoke in the next two months? Answers were recorded on a 7-point scale from 1 “I surely will not smoke” to 7 “I surely will smoke” [28], [9].

Grade level was used as representative of the students' age, ranging from first to fourth grade.

### Data analysis

The collected were analyzed quantitatively using Statistical Package for Social Science (SPSS). Between-group ANOVA and a hierarchical multiple regression were run to test hypotheses. For H3 and H4, which state a moderator role for grade level on the effects of parents' and peers' approval on intention to smoke, a traditional product term interaction analysis [29] was used.

We tested the variables for the basic assumption of normal distribution by looking at the Skewness and Kurtosis and for homoscedasticity via box plot. Since the dependent variable, intention to smoke, was not normally distributed, we use a logarithmic transformation to normalize the Skewness and Kurtosis. The transformed variable turned out to be normally distributed and was used in the analysis. The box plot for the new transformed variable supports the assumption of homoscedasticity. Missing data were less than 10%, and in the between-group ANOVA case were excluded analysis by analysis.

The two independent variables, perceived peers' and parents' approval, are included in the hierarchical multiple regression analysis via dummy variables. Since we have three categories for both (approve, neutral, not approve), we insert two dummy variables for each in the model, using “parents' disapproval” and “peers' disapproval” as reference group. The continuous moderator variable was centered and used for the interaction tested with traditional product-term analysis [29].

The hierarchical multiple regression was conducted in order to know how parents' and friends' approval and grade predicted intention to smoke; it suggests how much of the variance on the intention to smoke is explained by perceived parents' and friends' approval by controlling for sex and nationality, and how the variance changes when we consider grade as moderator variable. The coefficient for the product term reflects how much the mean difference in the predictor variables (parents' approval minus parents disapproval/peers' approval minus peers' disapproval) will change given one unit of change in the moderator continuous variable (centered grade level). The constructed model tests the contribution of each independent variable to the intention to smoke as well as the contribution of the model by considering first the control variables only and second all independent variables together, adding in a third step the interaction terms. In addition, regression models controlling for the parents' smoking behavior were computed, and the complete regression analysis was repeated for the group of students classified as smokers already.

## Results

ANOVA results demonstrate that the mean difference in intention to smoke is statistically significant for both predictor variables. For students whose parents approve smoking, the mean intention to smoke is higher by 2.92 scale points than for students whose parents disapprove smoking (*μ = 4.55 vs. μ* = 1.63). For students whose friends approve smoking the mean intention to smoke is 1.80 scale points higher than for those whose friends disapprove smoking (*μ* = 3.08 vs. *μ* =  1.28, Table 2). This means H1 and H2 are confirmed, and, based on the larger mean difference, that RQ1 can be answered: perceived parents' approval affects intention to smoke more strongly than perceived friends' approval.

**Table 2:** Intention to smoke by parents' and peers' approval.

Referent	Approval	Neutral	Disapproval	Mean difference	95% Confidence interval		p Value
					Lower	Upper	
Parents	4.55		1.63	2.92	2.31	3.52	<.001
		2.11	1.63	0.48	0.38	0.58	<.001
Friends	3.08		1.28	1.80	1.50	2.09	<.001
		2.01	1.28	0.73	0.63	0.82	<.001

*Note*. Cell entries are mean intention to smoke, on a scale from 1 to 7, with high averages indicating stronger intention.

Table 3, Model 1 shows that the demographic variables, namely gender and nationality, account for 0.2% of the variance in the hierarchical multiple regression. No difference in intention to smoke is found between girls and boys, while the adolescents with other than Swiss nationality are more likely to smoke than Swiss adolescents (B = .036).

**Table 3:** Hierarchical regression and interaction effect: the effect of parents', friends' approval and grade on the intention to smoke.

Predictor variables	Reference Group	Model 1			Model 2			Model 3		
		*B*	*beta*	*p Value*	*B*	*beta*	*p Value*	*B*	*beta*	*p Value*
Nationality		.036	.046	.001	.019	.025	.058	.020	.025	.056
Gender		−.005	−.011	.455	.003	.006	.639	.003	.005	.714
PA	PD				.307	.087	<.0001	.252	.071	<.0001
PN	PD				.075	.128	<.0001	.074	.127	<.0001
FA	FD				.250	.154	<.0001	.233	.143	<.0001
FN	FD				.103	.190	<.0001	.110	.203	<.0001
Grade					.036	.157	<.0001	.019	.080	.002
PA*Grade	PD							−.124	−.043	.030
PN*Grade	PD							.007	.017	.297
FA*Grade	FD							.043	.030	.045
FN*Grade	FD							.023	.078	.001
FA*PA								.267	.048	.010
FA* PA*Grade								.052	.012	.537
***(R^2^)/(Incr. R^2^)***		(.002)			(.129)			(.005)		

*Note.* PA = parents' approval; PD = parents' disapproval; PN = parents neutral; FA = friends' approval; FD = friends' disapproval; FN = friends neutral; Grade = grade centered variable; n = 5657.

Model 2 (Table 3) shows that the independent variables together, namely parents' and peers' approval and grade level, account for 12.9% of the variance, and thus have a moderate effect on the intention to smoke [30]. The variance in Model 2 significantly increases to 14.9% (not shown) when parents' smoking is introduced in the model, keeping the effect of the independent variables on the intention to smoke at a moderate level [30]. The introduction of interaction terms in the regression (Model 3) contributes to the variance in intention to smoke an additional 0.5%. (0.6% for the model with parents' smoking as control variable).

The regression confirms the relationship between parents' and friends' approval and intention to smoke and thus also confirms H1 and H2. School grade significantly moderates the relationship in opposite ways for the two predictor variables, confirming H3 and H4: as adolescents get older the influence of parents' approval declines and the effect of peers' approval increases. The significance levels of the product terms shows that this is true for the interactions between parents' approval and grade, both friends' approval and neutrality and grade, but not for parents' neutrality and grade. The latter means that grade does not affect the differential influence on intention to smoke of students' perception of parents' disapproval over perceived parents' neutrality.

Parents' smoking significantly contributes to increase the intention to smoke (not shown). If parents are perceived as approving adolescents' smoking and they smoke themselves, their children's intention to smoke will be higher than their counterparts' whose parents disapprove smoking and are non-smokers, but the effect of parents approval decrease as the adolescents get older (B = −.150). If parents' smoking is introduced in the regression, the relationship between students' intention to smoke and their perception of their friends' position (approval vs. disapproval) becomes insignificant.

Since 23.7% of the adolescents in the survey were classified as smokers, we conducted a similar hierarchical regression separately for the group of smokers (not shown). The results are similar in many respects: the influence of the adolescents' perception of parents' approval (B = −.207), the fact that the former is stronger than the latter (B = .037, Sig. 070 for friends' approval), the influence of parents' smoking as increasing the effect of perceived parents' approval on the intention to smoke.

In one respect, however, the results of the smokers among the adolescents differ from the results for the total sample. The mean difference in intention to smoke between students who perceive parents' approval and those who perceive that parents disapprove increases among smoking adolescents as they get older (B = .481), while the same difference decreased when the total sample was considered. This means that adolescents' perception of parents' approval increases the intention to smoke, and this effect increases with age for adolescents who already smoke but decreases for nonsmoking youths.

## Discussion

The goal of the study was to investigate how adolescents' intention to smoke was influenced by their perception of their parents' and peers' approval of smoking. We expected both perceived parents' and peers' approval to affect adolescents' smoking intention, but with a different strength depending on the students' age. The findings support the hypotheses and are consistent with the existing literature. Parents' and peers' injunctive norms (i.e. parents' and peers' approval) are predictors of adolescents' intention to smoke [7], [8], [9]. Moreover, the obtained results have the merit of including age as moderator variable. Other strengths of the paper are the use of a large sample of adolescents, a remarkably high response rate and a population that it is not usually presented in international literature of tobacco control.

Results are inconsistent with the literature that states parents' and peers' social norms have similar effects [10], [11] and with studies suggesting a stronger influence of peers [12]. The role of peers' influence is supported: peers are shown to be important components of adolescents' decisions to perform a behavior [7], [8], [9], [10], [11], [12], and they become more and more important as the adolescent gets older and independent, and as parental influence declines [31]. In fact, our findings demonstrate that the effect of adolescents' perception of parents' disapproval wanes as they get older. This confirms the theory of Sawyer and Stevenson [7].

Furthermore as result of the ANOVA and of the interaction analysis, parents and peers are predictors of adolescents' smoking intention, but parents' injunctive norms (i.e. parents' smoking approval and disapproval) influence smoking intention more strongly than peers' norms. Although the effect of parents' injunctive norms decreases with adolescents' age, parents' opinion still continues to affect the intention more than peers' injunctive norms. This supports previous research stating that adolescents are less likely to smoke if they perceive they parents react negatively to such behavior [13], [16], [17]. In that sense, parental behavior, in terms of monitoring and controlling their children's tobacco use and in terms of communicating about it [32], [33] should be considered an important factor in preventing tobacco consumption and initiation. Higher levels of parental monitoring, adolescent attachment to parents, and parent-adolescent communication about tobacco use all have been found to be inversely related to adolescents' tobacco use [34], [35]. Empirical evidence shows the effectiveness of different tobacco prevention and cessation programs and measures. Media campaigns, smoke-free legislation, taxation, ban on tobacco advertising and sales are crucial measures in the prevention of adolescents' smoking [36]. At the national level, the Swiss Confederation has used several measures for preventing smoking – i.e. increasing tobacco taxation, strengthening warnings and creating the Tobacco Control Fund. At the cantonal level, in Ticino, the following regulatory measures against smoking were implemented: limitations of tobacco advertising on radio and television; ban of advertising tobacco products on public soil and private places if they can be seen from the street; smoking bans in public places for protecting people from passive smoking; ban on tobacco sale and distribution and its derivatives to young people under 18 years of age. The legislative measures were complemented by numerous media campaigns and other national and cantonal projects that were successful in raising the awareness of specific target groups [37].

Despite the evident effectiveness of the implemented, and in addition to the existing measures, our results—the first to do so coming from Ticino—support the implementation of smoking prevention programs based on parent-child communication. The transmission/communication of disapproval of a behavior from parent to child is part of the communication process between parents and adolescents, and the manner in which information is related influences the adolescents' perception with respect to the subject under discussion [32], [33]. Past research suggests that communication style is associated with a reduced risk behavior and substance use [38], and high levels of parent-child affection ties or connectedness and parental disapproval have been shown to be protective factors for many adolescents health risk behaviors, including smoking [26], [39].

Even though some research shows evidence that parent-adolescent discussions about rules on smoking and drinking and parent control increase the use of these substances [40], [41], another part of the literature strongly supports that family communication and communication style are associated with reduced substance use [32], [33]. Parent-child communication techniques were also developed for parents who smoke, and they have been shown to be effective for this group of people. And they have a positive effect on adolescents' smoking also in the case one or both parents smoke [33], [40]. Parents are viewed as change agents who are valuable sources of information and advice to shape the behavior of their children [42]; their intervention and their communication style play a crucial role in reducing the risk for their children to become smokers in early adolescence. Family-based interventions that are brief, theory-based, and focused on risk and protective factors in the family and that target parenting competencies, training skills, and affective qualities of the parent-adolescent relationship can have a long-term protective effect on adolescents' tobacco use [43]. In line with the existing literature and with our finding supporting the importance of parents' social norms in early adolescence, we strongly suggest tobacco prevention programs targeting parent-adolescent communication be developed, that is: aiming at teaching parents to better communicate with their children about their feelings about tobacco. The parent-child communication techniques should be more effective if they consider the different needs and characteristic of the target group, i.e. age, parents' smoking status and adolescents' smoking status.

### Limitations and implications

First of all, that is the limitation of this study that we explored the adolescents' point of view without including the parents' perception. A comparison with parents' perception should be interesting.

Second, parental communication factors are not considered in the analysis. It should be important to analyze the relation between adolescents' perception of parents' norms and family communication factors, namely parents' communication style, frequency of communication and contents. This will yield additional information concerning tobacco use intention and will help to construct appropriate prevention programs improving family communication.

Finally, but not least, the cross-sectional methodology and measures of parent's and peers' smoking approval as perceived by adolescents are the most important weaknesses of the paper.

## Supporting Information

Survey S1 **Tabacco e Giovani (Tobacco and Adolescents).**
(DOCX)
