# Supplementary material for: Influence of Perceived Parent and Peer Endorsement on Adolescent Smoking Intentions: Parents Have More Say, But Their Influence Wanes as Kids Get Older
Source: PLoS One. 2014 Jul 3;9(7):e101275. doi: 10.1371/journal.pone.0101275 (PMC4081496; doi:10.1371/journal.pone.0101275)
Supplement: Survey S1 — Tabacco e Giovani (Tobacco and Adolescents). (DOCX) [file pone.0101275.s001.docx]

| 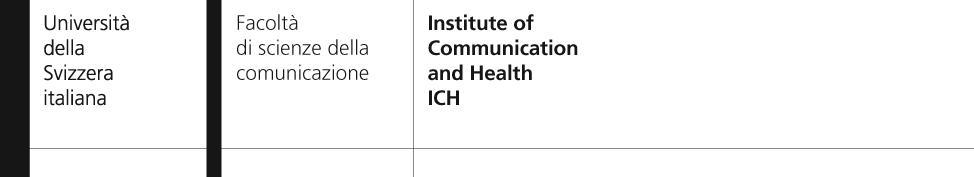  **Associazione Svizzera**  **non Fumatori** |
| --- |
| Questionario |
| **“Tabacco e giovani”** |
| Indagine sul consumo di tabacco  fra i giovani nelle scuole medie ticinesi |
|  |
| **USI - ICH/ASN** |
| **2011** |

|  |
| --- |

**Tabacco e giovani**

**Indagine sul consumo di tabacco fra i giovani delle scuole medie ticinesi.**

| Scuola: |  |
| --- | --- |
| Classe: |  |
|  |  |

**Istruzioni**

Il questionario verrà compilato in forma anonima e nessuno potrà venire a conoscenza della

tua identità, dunque puoi esprimerti in piena libertà.

Ti preghiamo di leggere attentamente le domande e segnare con una crocetta le tue

risposte.

**Consumo di tabacco**

1. Dopo aver visto una pubblicità di un McDonald’s, ti è mai venuta voglia di mangiare un hamburger?
   - Sì
   - No
2. Hai già fumato?
   - Sì
   - No **(VAI ALLA DOMANDA 10 SU PAGINA 3)**
3. A che età hai fumato la prima sigaretta?
   - Prima dei 7 anni
   - A 7 – 8 anni
   - A 9 – 10 anni
   - A 11 – 12 anni
   - A 13 – 14 anni
   - A 15 – 16 anni
   - A 17 anni
4. Quante volte, nel corso della tua vita, hai già fumato una sigaretta?
   - Nessuna volta
   - 1 - 2 volte
   - 3 – 5 volte
   - 6 – 9 volte
   - 10 – 19 volte
   - 20 – 39 volte
   - 40 volte o più
5. Attualmente fumi
   - Ogni giorno
   - Una volta la settimana
   - Meno di una volta la settimana
   - Attualmente non fumo
6. Se attualmente fumi, quante sigarette consumi?
   - Più di un pacchetto
   - Un pacchetto
   - Da 13 a 20 sigarette
   - Da 8 a 12 sigarette
   - Da 3 a 7 sigarette
   - Meno di 3 sigarette
   - Attualmente non fumo
7. Chi ti ha offerto la prima sigaretta?
   - Mio padre/mia madre
   - Mio fratello/mia sorella
   - Un amico/un’amica
   - Un adulto/un’adulta (non un genitore)
   - Nessuno, me la sono procurata da solo
8. Come ti procuri di solito le sigarette?

**(Più risposte sono possibili)**

- - Le compro in un negozio o al chiosco
  - Le compro al distributore automatico
  - Me le danno mio fratello/mia sorella
  - Me le danno mio padre/ mia madre
  - Me le danno i compagni/le compagne
  - Le prendo dove mi capita
  - Altro **(specificare)** ………………………………………………………………………………

1. I tuoi genitori sanno che attualmente fumi o che hai già provato una sigaretta?
   - Sì, lo sanno sicuramente
   - Non lo sanno direttamente, ma lo pensano
   - Non so se lo sanno
   - Non lo sanno sicuramente
2. Adesso pensa a te stesso: fra due mesi, hai intenzione di fumare oppure no?

**Su una scala da 1 a 7 segna il punto corrispondente alla tua risposta. Se sei molto sicuro/a che intendi fumare o se sei molto sicuro/a che non intendi fumare sicuramente, segna una delle due estremità della scala. Se non sei tanto sicuro/a, scegli il punto intermedio più adatto. Ricordati che il punto 4 indica: “indifferente”.**

Non intendo fumare sicuramente

Intendo fumare sicuramente

|  | |  |  |  |  |  |  |  |  |  |  |  |  |  |
| --- | --- | --- | --- | --- | --- | --- | --- | --- | --- | --- | --- | --- | --- | --- |
|  |  | 1 |  | 2 |  | 3 |  | 4 |  | 5 |  | 6 |  | 7 |

1. Cosa pensano i tuoi genitori dei ragazzi della tua età che fumano regolarmente?

**(Solo una risposta è possibile)**

- - Sono contrari
  - Sono indifferenti
  - Li approvano
  - Non lo so

1. Tuo padre fuma?
   - Sì
   - No
   - Non fuma più
2. Tua madre fuma?
   - Sì
   - No
   - Non fuma più
3. I tuoi compagni e/o amici fumano regolarmente?

- Tutti
- La maggior parte
- Alcuni
- Pochi
- Nessuno fuma

1. Quanto spesso i tuoi compagni e/o amici fumano?
   - Ogni giorno
   - Una volta la settimana
   - Meno di una volta la settimana
   - Nessuno fuma
2. Fra i tuoi compagni e/o amici fumano di più i ragazzi o le ragazze?

- I ragazzi fumano di più
- Le ragazze fumano di più
- Non c’è differenza fra ragazzi e ragazze
- Nessuno fuma

1. Cosa pensi dei tuoi compagni e/o amici che fumano?

- Sono contrario/a
- Sono indifferente
- Li approvo

1. Cosa pensano i tuoi compagni e/o amici dei ragazzi che fumano?

**(Solo una risposta è possibile)**

- - Sono contrari
  - Li approvano
  - Alcuni sono contraria e altri li approvano
  - Sono indifferenti

1. Secondo te, perché i giovani fumano? **(Più risposte sono possibili)**
   - Per imitare gli amici
   - Per imporsi agli altri
   - Per curiosità
   - Per piacere
   - Per seguire l’esempio degli adulti
   - Perché è facile procurarsi le sigarette
   - Per farsi “belli” davanti agli altri
   - Altro……………………………………………………………………………………..
2. Indica quanto sei soddisfatto/a della relazione con tua madre

- Molto soddisfatto/a
- Soddisfatto/a
- Indifferente
- Non soddisfatto/a
- Per niente soddisfatto/a
- Non ho nessuna relazione

1. Indica quanto sei soddisfatto/a della relazione con tuo padre

- Molto soddisfatto/a
- Soddisfatto/a
- Indifferente
- Non soddisfatto/a
- Per niente soddisfatto/a
- Non ho nessuna relazione

1. Quale di queste affermazioni descrive meglio quanto spesso hai parlato con i tuoi genitori della problematica del fumo? (Si intende una vera discussione, non solo menzionare l’argomento).

**(Scegli una sola risposta che descrive meglio la tua situazione)**

- - Non ho mai parlato con i miei genitori del fumo
  - Non abbiamo mai fatto una vera discussione ma ogni tanto ho captato quello che loro pensano riguardo quest’argomento
  - Non abbiamo mai fatto una vera discussione ma hanno solo accennato all’argomento
  - Ho parlato con i miei genitori del fumo circa 1 o 2 volte (si intende una vera discussione)
  - Ho parlato con i miei genitori del fumo da 3 a 5 volte (si intende una vera discussione)
  - Ho parlato con i miei genitori del fumo da 6 a 10 volte (si intende una vera discussione)
  - Ho parlato con i miei genitori del fumo più di 11 volte (si intende una vera discussione)

1. Indica se ti sei mai trovato/a in una di queste situazioni:

“Non sono mai stato triste”
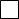
 Vero
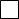
 Falso

“Non ho mai criticato altre persone”
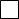
 Vero
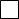
 Falso

“Non ho mai discusso con gli altri”
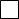
 Vero
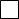
 Falso

1. Sono i tuoi genitori che hanno iniziato a parlarti dell’argomento fumo o sei stato/a tu?

**(Scegli una sola risposta che descrive meglio la tua situazione)**

- - Nella maggior parte dei casi hanno preso loro l’argomento
  - Alcune volte lo hanno fatto loro e altre io
  - Nella maggior parte dei casi l’ho fatto io
  - Non ho mai parlato con i miei genitori del fumo

1. Indica le ragioni per cui i tuoi genitori o tu avete iniziato a parlare della problematica del fumo
   - Hanno scoperto che fumo
   - Ho comunicato loro che fumo
   - Hanno saputo o visto che i miei amici/le mie amiche fumano
   - Solo perché i miei genitori hanno voluto affrontare o accennare all’argomento
   - Perché io volevo avere delle informazioni sul fumo
   - Non ho mai parlato con i miei genitori del fumo
   - Altro **(Specificare)** …………………………………………………………………………………….
2. Quando hai parlato con i tuoi genitori riguardo al fumo, loro hanno cercato di:

**(Più risposte sono possibili)**

- - Dissuaderti dal fumare altrimenti avresti avuto una punizione
  - Dissuaderti dal fumare dandoti delle motivazioni
  - Consigliarti semplicemente di non fumare
  - Non ti hanno detto niente
  - Per loro è indifferente
  - Non ho mai parlato con i miei genitori dell’argomento fumo

1. Cosa ti hanno detto i tuoi genitori delle conseguenze che può avere il fumo?

**(Più risposte sono possibili)**

- - Che fumare fa male
  - Che fumare nuoce o da fastidio agli altri
  - Che fumare costa caro
  - Che è molto difficile smettere una volta che si comincia
  - Che i tuoi vestiti o la tua stanza puzzano quando fumi
  - Che fumare ti potrebbe portare all’uso di altre sostanze che provocano dipendenza
  - Nessuna delle precedenti
  - I miei genitori non mi hanno mai parlato delle conseguenze del fumo
  - Altro **(Specificare)** …………………………………………………………………………………

1. Pensi che i tuoi genitori abbiano fatto bene a metterti in guardia sulle conseguenze del fumo?
   - Sì
   - No
   - Non lo so
   - I miei genitori non mi hanno mai parlato delle conseguenze del fumo
2. Le informazioni che ti hanno dato i tuoi genitori sul fumo, hanno avuto un impatto sul tuo comportamento riguardo al fumare?

**(Solo una risposta è possibile)**

- Non fumo, ma sono state utili
- Non fumo, e non mi sono servite a niente
- Sì, ho smesso di fumare
- Sì, ma continuo a fumare ugualmente
- Sì, sto cercando di smettere
- Sì, fumo di meno
- No, continuo a fumare
- Indifferente
  - Non ho mai parlato con i miei genitori del fumo

| **Di seguito trovi alcune affermazioni. Su una scala da 1 a 7, segna con una crocetta se e quanto sei d’accordo oppure no con l’affermazione. Se sei completamente d’accordo o se sei completamente in disaccordo, segna le rispettive estremità. Se sei d’accordo o in disaccordo parzialmente, scegli il punto intermedio più adatto. Il punto 4 indica “indifferente”. Per esempio:**  **Significa che sei completamente in disaccordo** | | | | | | | | | | | | | | | | | |  |  |  |  |  |  |
| --- | --- | --- | --- | --- | --- | --- | --- | --- | --- | --- | --- | --- | --- | --- | --- | --- | --- | --- | --- | --- | --- | --- | --- |
| Disaccordo | |  |  |  |  |  |  |  |  |  |  |  |  |  | D'accordo |  |  |  |  |  |  |  |  |
|  |  | 1 |  | 2 |  | 3 |  | 4 |  | 5 |  | 6 |  | 7 |  |  |  |  |  |  |  |  |  |

**Significa che sei d’accordo un po`**

| Disaccordo | |  |  |  |  |  |  |  |  |  |  |  |  |  | D'accordo |
| --- | --- | --- | --- | --- | --- | --- | --- | --- | --- | --- | --- | --- | --- | --- | --- |
|  |  | 1 |  | 2 |  | 3 |  | 4 |  | 5 |  | 6 |  | 7 |  |

1. Il fumo degli altri mi da fastidio!

| Disaccordo | |  |  |  |  |  |  |  |  |  |  |  |  |  | D'accordo |
| --- | --- | --- | --- | --- | --- | --- | --- | --- | --- | --- | --- | --- | --- | --- | --- |
|  |  | 1 |  | 2 |  | 3 |  | 4 |  | 5 |  | 6 |  | 7 |  |

1. Il fumo è dannoso per la salute!

| Disaccordo | |  |  |  |  |  |  |  |  |  |  |  |  |  | D'accordo |
| --- | --- | --- | --- | --- | --- | --- | --- | --- | --- | --- | --- | --- | --- | --- | --- |
|  |  | 1 |  | 2 |  | 3 |  | 4 |  | 5 |  | 6 |  | 7 |  |

1. Sono a mio agio a parlare con i miei genitori dell’argomento fumo!

| Disaccordo | |  |  |  |  |  |  |  |  |  |  |  |  |  | D'accordo |
| --- | --- | --- | --- | --- | --- | --- | --- | --- | --- | --- | --- | --- | --- | --- | --- |
|  |  | 1 |  | 2 |  | 3 |  | 4 |  | 5 |  | 6 |  | 7 |  |

1. Parlare con i miei genitori della problematica del fumo è importante!

| Disaccordo | |  |  |  |  |  |  |  |  |  |  |  |  |  | D'accordo |
| --- | --- | --- | --- | --- | --- | --- | --- | --- | --- | --- | --- | --- | --- | --- | --- |
|  |  | 1 |  | 2 |  | 3 |  | 4 |  | 5 |  | 6 |  | 7 |  |

1. Hai mai visto questo logo?

- - Sì
  - No

1. È la prima volta che vedi questa campagna?

- - Sì
  - No

**Adesso siamo arrivati alla fine, solo qualche ultima domanda!**

1. Sesso

- Maschio
- Femmina

1. Nazionalità

____________________________________________________

1. Data di nascita

| Mese | Anno |
| --- | --- |
|  |  |

Ti ricordiamo che le risposte verranno trattate in forma anonima.

**Ti ringraziamo per la tua preziosa collaborazione e per il tempo dedicatoci!**

Se sei interessato/a a conoscere i risultati del sondaggio puoi contattarci al seguente
indirizzo e-mail:

francesca.scalici@usi.ch

**collegamenti utili:**

<http://www.nonfumatori.ch/>

[www.usi.ch](http://www.usi.ch)

<http://www4.ti.ch/decs/ds/uim/ufficio/>
